# Association of Blood Selenium Levels with Diabetes and Heart Failure in American General Adults: a Cross-sectional Study of NHANES 2011–2020 pre

**DOI:** 10.1007/s12011-023-03933-4

**Published:** 2023-11-23

**Authors:** Chongyang Zhang, Qingjia Zeng, Xinyao Liu, Qile He, Jinyao Zhang, Shanshan Zhao, Hongpu Hu

**Affiliations:** Institute of Medical Information/Library, Chinese Academy of Medical Sciences & Peking Union Medical College, Beijing, 100020 China

**Keywords:** Selenium, Diabetes, Heart failure, Risk factors

## Abstract

Selenium is an essential trace element closely related to human health; however, the relationship between blood selenium levels, diabetes, and heart failure remains inconclusive. Therefore, this study aimed to explore the relationship between blood selenium levels and the prevalence of diabetes as well as heart failure in American general adults aged 20 years or older. This study utilized data from four survey cycles from NHANES 2011–2020 pre. Blood selenium levels were considered as both a continuous variable and quartiles, and logistic regression was employed to investigate the associations between blood selenium levels with diabetes and heart failure. Nonlinear relationships were examined by restricted cubic spline regression. The analysis included a total of 16311 participants aged 20 years or older. After adjustment for all potential confounder, we found when the blood selenium levels increased by 10 ug/L, the average risk of diabetes increased by 4.2% (95% CI: 1.5%, 7.0%), and the average risk of heart failure decreased by 5.0% (95% CI: 0.1%, 9.8%). In addition, compared with the lowest reference group, blood selenium levels were significantly positively associated with risk of diabetes in participants in the fourth quartile (OR=1.458, 95% CI: 1.173, 1.812), while significantly negatively associated with the risk of heart failure in participants in the second, third and fourth quartiles (Q2, OR=0.677, 95% CI: 0.471, 0.974) (Q3, OR=0.609, 95% CI: 0.426, 0.870) (Q4, OR=0.653, 95% CI: 0.443, 0.961). There was a nonlinear and reverse L-shaped association between blood selenium and diabetes, while a negative dose-response association between blood selenium and heart failure. Furthermore, the association between blood selenium levels and heart failure was more pronounced in participants with poor glycemic control, rather than diabetic patients. High blood selenium levels may be positively related to diabetes, while low blood selenium levels may be associated to heart failure. Appropriate blood selenium levels may help prevent diabetes and heart failure.

**Supplementary Information:**

The online version contains supplementary material available at 10.1007/s12011-023-03933-4.

## Introduction

Diabetes, a chronic metabolic disease, is typified by hyperglycemia that may cause damage to various organs systems. In 2021, the global prevalence of diabetes among people aged 20–79 years was estimated to be 10.5% (equivalent to 536.6 million people), with 6.7 million adults died from diabetes or its complications, accounting for 12.2% of all causes of death [1]. In the USA, the prevalence of type 2 diabetes exceeded the global average by about 25%, with its disease burden consistently increasing [2].

Uncontrolled hyperglycemia can induce insulin resistance, oxidative stress, inflammatory reactions, disturbances in glucose and lipid metabolism, and a series of complications, all of which contribute significantly to disability and increased mortality in diabetes. Many studies have shown that poor glycemic control was closely associated with an increased risk of heart failure [3, 4]. Heart failure, the final stage of numerous cardiovascular diseases, is a clinical syndrome resulting from abnormal cardiac structure or function, predominantly due to blood circulation disruptions [5]. This non-fatal cardiovascular disease affects approximately 64 million individuals globally and its incidence is expected to surge with population aging and improved diagnostic techniques [6]. Consequently, heart failure poses a significant threat to human life expectancy and health, as well as placing a heavy burden on healthcare systems. Therefore, it is crucial to clarify the potential risk factors and detrimental effects of diabetes and heart failure.

Selenium is an essential trace element for humans and is integral to the function of glutathione peroxidase [7]. In organisms, selenium primarily exists in different organic and inorganic compounds. Its health impacts can vary based on factors such as dietary intake, environmental exposure, and metabolic status [8]. In recent years, studies on the relationship between selenium and diabetes and heart failure have gradually increased, but different biomarkers were used and the results have been inconsistent. While certain research suggests that excessive selenium accumulation in the body is positively correlated with type 2 diabetes [9], other studies find no significant associations [10]. Moreover, it has been observed that serum selenium is U-shaped related to all-cause and cardiovascular disease mortality [11]. It is also worth noting that the association between selenium status and disease may also vary among different populations [12]. Selenium deficiency has been linked to higher all-cause mortality rates and hospital readmissions for heart failure (HR 1.23; 95% CI 1.06–1.42) [13]. Given these varying results, there is a pressing need for comprehensive research on the impact of selenium on diabetes and heart failure.

However, the existing evidence on the relationship between blood selenium levels, diabetes, and heart failure remains inconclusive and requires further investigation. To address this knowledge gap, we utilized data from National Health and Nutrition Examination Survey (NHANES) 2011–2020 pre to investigated the associations between blood selenium levels with diabetes and heart failure in general American adults aged 20 years and over. We also explored the associations between different participants, identified high-risk groups, which could provide more precise references for diabetes and heart failure prevention.

## Method

### Data Source and Study Population

The NHANES survey, conducted by the American National Center for Health Statistics biennially since 1999, aimed to collect health and nutritional information about the civilian population using a stratified, complex, multi-stage, and probabilistic approach. However, the onset of the COVID-19 pandemic in March 2020 compromised the representativeness of the data from that period. To address this, data from 2019 to March 2020 were combined with data from the NHANES 2017–2018 cycle to create a nationally representative sample of NHANES 2017-March 2020 pre-pandemic data [14, 15]. Participants in the survey are assigned specific weights based on their sampling probabilities and non-response rates to ensure accurate representation of the population. This approach facilitates the identification of meaningful survey results and enhances the validity and reliability of the collected data. In this study, we included all adults aged 20 years or older who participated NHANES 2011–2012, 2013–2014, 2015–2016, and 2017-March 2020 surveys, with blood selenium concentrations being recorded since 2011. Ethical review was conducted for the survey procedures by the Centers for Disease Control and Prevention (CDC) and all subjects provided informed consent. All methods employed in this study were conducted in strict accordance with relevant guidelines and regulations, please refer to the NHANES dataset introduction for more details (https://www.cdc.gov/nchs/nhanes/index.htm).

**Fig. 1:**
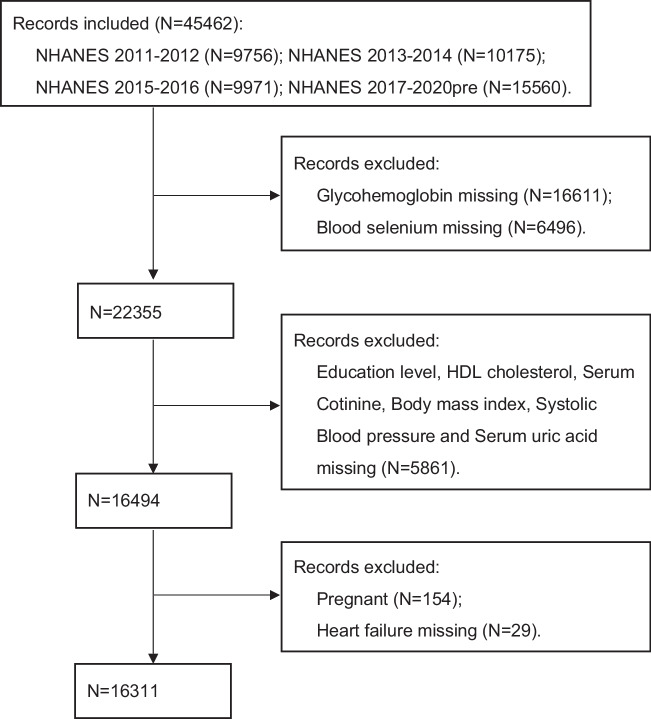
Sample selection flowchart

A total of 45462 subjects were initially included in the analysis. Participants were excluded based on the lack of glycohemoglobin (HbA1c) and blood selenium concentrations data (*N*=23107). Next excluded participants under the age of 20 and those with missing covariates data, such as education level, high-density lipoprotein cholesterol (HDL-C), serum cotinine, body mass index (BMI), systolic blood pressure and serum uric acid (*N*=5861). Then excluded those who were pregnant (*N*=154) and heart failure deficiency (*N*=29). Eventually, 16311 subjects were included in the analysis (Fig. 1).

### Blood Selenium Level Measurements

The biomarkers used to evaluate selenium status of the body include blood (whole blood, plasma, or serum), toenails, hair, and urine. In this study, we adopted the whole blood selenium as one of the best indicators for evaluating short-and-medium-term selenium exposure in the human body. Compared to other indicators, it provides a more precise representation of the aggregate selenium concentration. Additionally, it enables a comprehensive assessment of selenium distribution within different cellular components, enriching our understanding of how selenium is distributed and utilized in the organism. Moreover, whole blood selenium is less susceptible to external factors, enhancing its reliability and robustness as a biomarker for assessing selenium status [10, 16].

To ensure the accuracy and reliability of the results, rigorous quality assurance and quality control standards were followed for testing all laboratory samples in this study. Blood samples were collected and stored appropriately at a frozen temperature of -20°C until they were transported to the American National Center for Environmental Health for analysis. To carry out a uniform distribution of cellular components, the whole blood samples were diluted by combining 1 part sample + 1 part water + 48 parts of diluent, which consisted of substances such as Tetramethylammonium hydroxide (TMAH, 0.4% v/v), Triton X-100TM (0.05%), Ammonium pyrrolidine dithiocarbamate (APDC, 0.01%), ethanol (1%), etc. These diluents served several functions, including releasing selenium bound to red blood cells, reducing ionization suppression by the biological matrix, preventing clogging of the sample introduction system pathways, and facilitating the introduction of internal standards for analysis. The selenium concentrations in whole blood samples were determined using inductively coupled plasma mass spectrometry (ICP-DRC-MS). This method used methane (CH4, 99.999%) as the reaction gas to minimize multi-atomic interference during analysis and detect the strength of the ^80^Se^+^ ion. The limit of detection (LOD) for blood selenium concentration was 24.48 μg/L [10, 17].

### Outcomes

Diabetes was defined as (1) self-report of diabetes, currently taking glucose-lowering drugs, or using insulin; (2) fasting plasma glucose ≥126 mg/dL (7.0 mmol/L); and (3) glycohemoglobin (HbA1c) ≥6.5% [18]. Heart failure was defined as self-reported diagnosis of congestive heart failure by a doctor or other healthcare professional.

### Covariates

NHANES was conducted according to established protocols and trained personnel. The selection of covariates for this study was based on relevant literature, including factors such as age, gender, race, education level, PIR, tobacco exposure, BMI, physical activity, alcohol consumption, hypertension, hyperuricemia, total cholesterol (TC), HDL-C, albumin, creatinine, blood urea nitrogen (BUN) levels and heart failure/HbA1c [9, 11, 12, 19].

Tobacco exposure was determined by serum cotinine greater than 1ng/ml [20]. Participants were identified as physical activity if they participated in moderate or vigorous intensity activities (including recreational and work activities). Alcohol consumption was characterized as an average intake of greater than or equal to 2 alcoholic beverages per day for the past 12 months. Hypertension was clarified as self-reported hypertension, taking antihypertensive medication, or having a systolic blood pressure ≥140 mmHg and/or a diastolic blood pressure ≥90 mmHg [21]. Hyperuricemia was described as serum uric acid ≥ 416 μmol/L (7.0 mg/dL) in men or ≥ 357 μmol/L (6.0 mg/dL) in women [22]. High total cholesterol was elucidated as total cholesterol ≥ 5.173 mmol/L (200 mg/dl) [23]. Poor glycemic control was defined as HbA1c >7% [24].

### Statistical Analysis

Given the intricate and multi-stage sampling design of the NHANES, which includes nonresponse and oversampling, all analyses were performed using the weights from the blood selenium subsample [14]. Since PIR and alcohol consumption may be potential factors affecting glucolipid metabolism [25], multiple interpolation was employed to fill in the missing values of PIR and alcohol variables.

Descriptive statistics were used to summarize non-normally distributed continuous covariates through weighted median (weighted interquartile range), while categorical variables were presented with frequency (weighted percentages). To compare participants based on blood selenium levels, we applied the Kruskal-Wallis *H* test for continuous variables and the *χ*^2^ test for categorical ones.

Weighted multivariate logistic regression models were employed to estimate the odds ratio (OR) and the corresponding 95% confidence interval (CI) for the prevalence of diabetes or heart failure based on quartiles of blood selenium concentrations. Quartile-transformed blood selenium concentrations served as rank variables to explore the linear trend effect. When blood selenium was regarded as a continuous variable, OR and its 95% CI were reported for each 10 μg/L increment in the blood selenium levels. Both models were adjusted for covariates, model 1 was adjusted for demographic characteristics, including age, gender, race, education level and PIR. Model 2 was further adjusted for serum cotinine, BMI, physical activity, alcohol, hypertension. Model 3 was further adjusted for hyperuricemia, TC, HDL-C, albumin, creatinine, BUN, and further adjusted heart failure for diabetes and HbA1c for heart failure.

To investigate the potential nonlinear relationship between blood selenium levels and the risk of developing diabetes or heart failure, restricted cubic spline (RCS) regression models with four nodes (5th, 35th, 65th, and 95th percentile) were developed in addition[26]. We employed the likelihood ratio test to determine whether the relationship was non-linear, with *P*_*for non-linearity*_ ≤ 0.05 indicated a non-linear relationship.

To access the potential modifying effects of diabetes and poor glycemic control, subgroup analysis was performed to explore the associations between blood selenium levels and heart failure. Additionally, an interaction term was introduced into the models to examine potential interaction effects between blood selenium levels and covariates. Subsequently, subgroup analysis was conducted to evaluate interaction and the potential modifying effects of various important factors, including age, gender, education level, PIR, tobacco exposure, BMI, physical activity, alcohol consumption, hypertension, hyperuricemia, high total cholesterol (and heart failure) on the association of blood selenium levels with diabetes and heart failure [21, 27].

We conducted sensitivity analyses by adjusting for different covariates to test the robustness of our results. Sequentially, we excluded in turn the individuals those were taking selenium supplements, those aged over 80 years, interpolated data, as well as those who were currently taking glucose-lowering drugs or insulin.

All statistical analyses were performed using R software (version 4.2.1, https://www.r-project.org/). The level of statistical significance was set at *P*<0.05 for the two-sided test, and significance level of *P*<0.10 was used to determine the presence of interaction effects.

## Results

### Characteristics of the Participants

Table 1 summarized the baseline characteristics of the study participants categorized by quartiles of blood selenium levels. The analysis included a total of 16311 participants, with a median age of 48.00 [33.00, 61.00] years, and 49.2% of them were males. The weighted median (IQR) concentration of blood selenium was 191.62 (176.96, 207.22) μg/L, with a detection rate of Se was 100.00%. Among the participants, there were 3107 patients with diabetes, with a weighted prevalence of 14.3%, and 537 patients with heart failure, with a weighted prevalence of 2.4%. Males (*P*<0.001), Non-Hispanic White and other race individuals (*P*<0.001), those with PIR≥3.5 (*P*=0.006), without tobacco exposure (*P*<0.001), hyperuricemia (*P*=0.010), high total cholesterol levels (*P*<0.001), lower HDL-C levels (*P*=0.001), and poor glycemic control (*P*=0.006) were more likely to have higher blood selenium concentrations. There was no significant difference in the prevalence of diabetes among participants with different blood selenium levels (*P*=0.114). In contrast, the prevalence of heart failure was higher among participants in the first quartile of blood selenium levels (*P*<0.001).

**Table 1:** Characteristics of study participants by quartiles of blood selenium levels

Characteristics	Overall	Q1 (<176.96μg/L)	Q2 (176.96-191.62μg/L)	Q3 (191.62-207.22μg/L)	Q4 (>207.22μg/L)	*P*
Age/[M(Q1,Q3)] ^a^	48.00 [33.00, 61.00]	48.00 [33.26, 63.00]	47.00 [33.00, 61.00]	47.00 [33.00, 60.00]	48.00 [34.00, 61.00]	0.121
Gender/n (%)						**<0.001**
Male	8138(49.2)	2162(43.5)	1998(47.5)	1949(51.3)	2029(54.5)	
Female	8173(50.8)	2666(56.5)	2056(52.5)	1798(48.7)	1653(45.5)	
Race/n (%)						**<0.001**
Mexican American	1991(8.3)	480(7.3)	540(8.8)	481(8.7)	490(8.5)	
Other Hispanic	1722(6.5)	576(8.2)	447(6.8)	381(6.1)	318(5.1)	
Non-Hispanic White	6115(65.7)	1706(62.5)	1479(64.8)	1471(67.6)	1459(67.9)	
Non-Hispanic Black	3883(10.8)	1446(14.1)	962(11.2)	805(9.6)	670(8.2)	
Other Race	2600(8.7)	620(7.8)	626(8.4)	609(8.0)	745(10.3)	
Education level/n (%) ^a^						**0.002**
Less than 9th grade	1384(4.7)	442(5.8)	325(4.5)	301(4.5)	316(4.2)	
9-11 grade	1927(8.5)	639(10.0)	489(8.1)	408(7.9)	391(8.0)	
High school or equivalent	3705(23.0)	1178(25.2)	909(23.6)	836(21.9)	782(21.4)	
Some college or AA degree	5128(31.7)	1482(29.0)	1302(33.0)	1195(32.6)	1149(32.2)	
College or above	4167(32.0)	1087(30.0)	1029(30.8)	1007(33.0)	1044(34.2)	
PIR/n (%) ^a^						**0.006**
≤1.30	5091(21.5)	1678(24.7)	1219(20.4)	1098(20.2)	1096(20.6)	
1.31-3.49	6083(35.5)	1764(35.3)	1519(35.7)	1428(35.9)	1372(35.2)	
≥3.50	5137(43.0)	1386(39.9)	1316(43.9)	1221(43.9)	1214(44.2)	
Serum cotinine/n (%)						**<0.001**
<1.00 ng/ml	11755(73.1)	3288(69.9)	2918(72.1)	2762(74.7)	2787(75.8)	
≥1.00 ng/ml	4556(26.9)	1540(30.1)	1136(27.9)	985(25.3)	895(24.2)	
BMI/n (%) ^a^						**0.004**
<25.00	4541(27.8)	1429(31.0)	1103(27.6)	1028(26.6)	981(26.0)	
25.00-29.99	5256(33.2)	1444(30.7)	1306(33.5)	1207(32.9)	1299(35.8)	
≥30.00	6514(39.0)	1955(38.3)	1645(39.0)	1512(40.5)	1402(38.2)	
Physical activity/n (%)						**0.044**
No	5063(25.6)	1641(27.9)	1254(25.7)	1076(24.4)	1092(24.7)	
Yes	11248(74.4)	3187(72.1)	2800(74.3)	2671(75.6)	2590(75.3)	
Alcohol consumption /n (%)						0.902
No	6350(36.9)	1898(36.3)	1557(36.9)	1456(37.6)	1439(36.8)	
Yes	9961(63.1)	2930(63.7)	2497(63.1)	2291(62.4)	2243(63.2)	
Hypertension/n (%)						0.300
No	9291(62.9)	2673(62.7)	2345(64.1)	2187(63.6)	2086(61.1)	
Yes	7020(37.1)	2155(37.3)	1709(35.9)	1560(36.4)	1596(38.9)	
Hyperuricemia/n (%)						**0.010**
No	13215(82.2)	3966(84.0)	3296(82.1)	3049(82.7)	2904(80.0)	
Yes	3096(17.8)	862(16.0)	758(17.9)	698(17.3)	778(20.0)	
High total cholesterol/n (%)						**<0.001**
No	10433(62.6)	3487(71.4)	2599(64.3)	2353(62.1)	1994(52.8)	
Yes	5878(37.4)	1341(28.6)	1455(35.7)	1394(37.9)	1688(47.2)	
Diabetes/n (%)						0.114
No	13204(85.7)	3900(85.5)	3331(86.7)	3044(86.7)	2929(84.2)	
Yes	3107(14.3)	928(14.5)	723(13.3)	703(13.3)	753(15.8)	
Poor glycemic control/n (%)						**0.006**
No	15011(94.6)	4479(94.8)	3778 (95.5)	3443 (94.8)	3311 (93.2)	
Yes	1300(5.4)	349 (5.2)	276 (4.5)	304 (5.2)	371 (6.8)	
Heart failure/n (%)						**<0.001**
No	15774(97.6)	4592(96.2)	3930(97.9)	3662(98.2)	3590(98.1)	
Yes	537(2.4)	236(3.8)	124(2.1)	85(1.8)	92(1.9)	
TC/[mmol/L, M(Q1,Q3)] ^a^	4.86 [4.19, 5.56]	4.63 [4.03, 5.30]	4.84 [4.16, 5.53]	4.86 [4.24, 5.59]	5.12 [4.42, 5.90]	**<0.001**
HDL-C/[mmol/L, M(Q1,Q3)] ^a^	1.32 [1.09, 1.63]	1.37 [1.11, 1.66]	1.34 [1.11, 1.63]	1.29 [1.09, 1.60]	1.29 [1.06, 1.58]	**0.001**
Albumin/[g/dL, M(Q1,Q3)] ^a^	4.30 [4.00, 4.50]	4.20 [3.90, 4.40]	4.30 [4.00, 4.50]	4.30 [4.10, 4.50]	4.30 [4.10, 4.60]	**<0.001**
Creatinine/[mg/dL, M(Q1,Q3)] ^a^	0.85 [0.72, 0.99]	0.84 [0.71, 0.99]	0.84 [0.72, 0.99]	0.85 [0.72, 0.99]	0.85 [0.72, 0.99]	0.233
BUN/[mg/dL, M(Q1,Q3)] ^a^	13.00 [11.00, 17.00]	13.00 [11.00, 17.00]	14.00 [11.00, 17.00]	13.00 [11.00, 17.00]	13.00 [11.00, 16.00]	0.538

M (Q1, Q3), Weighted Median (the weighted 25th percentiles, the weighted 75th percentiles); n (%), number (weighted percentage); a, Kruskal-Wallis *H* test; Bold formatting indicated statistically significant differences

*PIR* ratio of family income to poverty, *BMI* body mass index, *TC* total cholesterol, *HDL-C* high-density lipoprotein cholesterol, *BUN* blood urea nitrogen, *FPG* fasting plasma glucose, *HbA1c* glycohemoglobin

### Associations of Blood Selenium Levels with Diabetes

**Fig. 2:**
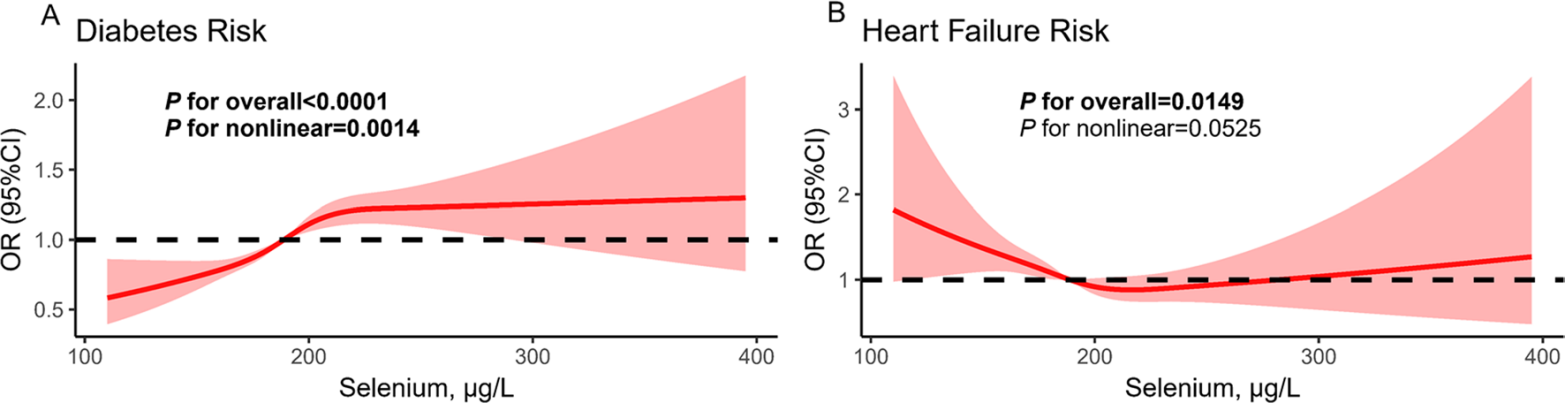
Restricted cubic spline regression between associations of blood selenium levels with diabetes and heart failure. Note: (**A**), diabetes; (**B**), heart failure

Blood selenium levels were significantly positively associated with risk of diabetes in participants in the fourth quartile compared to those in the first quartile (OR=1.458, 95% CI: 1.173, 1.812), with a significant positive trend observed even after adjusting for all potential confounders (*P* for trend=0.001). Each 10ug/L increase in blood selenium levels was associated with a 4.2% (95% CI: 1.5%, 7.0%) increase in the risk of diabetes (Table 2). RCS regression revealed a significant non-linear positive dose-response and reverse L-shaped association between blood selenium levels and risk of diabetes (*P*_overall_ <0.001, *P*_non-linear_=0.001, Fig. 2A).

**Table 2:** Associations of blood selenium levels with diabetes

Selenium	Q1 (<176.96μg/L)	Q2 (176.96-191.62μg/L)	Q3 (191.62-207.22μg/L)	Q4 (>207.22μg/L)	*P* for trend	OR (95% CI) for per 10μg/L increment
Median,ug/L	166.22	184.50	198.96	220.33		
Model1	ref(1.000)	0.992(0.822,1.198)	1.044(0.869,1.254)	**1.248(1.028,1.515)**	**0.020**	1.023(0.996,1.050)
Model2	ref(1.000)	0.980(0.800,1.200)	1.008(0.833,1.220)	**1.243(1.013,1.525)**	**0.034**	1.024(0.997,1.053)
Model3	ref(1.000)	1.030(0.841,1.262)	1.092(0.893,1.336)	**1.444(1.164,1.791)**	**0.001**	**1.041(1.014,1.069)**
Model4	ref(1.000)	1.043(0.849,1.280)	1.103(0.902,1.350)	**1.458(1.173,1.812)**	**0.001**	**1.042(1.015,1.070)**

OR (95% CI) were calculated with the multivariate logistic regression model; Bold formatting indicated statistically significant differences

Model 1 was adjusted for demographic characteristics, including age, gender, race, education level and PIR. Model 2 was further adjusted for serum cotinine, BMI, activity, alcohol, hypertension. Model 3 was further adjusted for hyperuricemia, TC, HDL-C, albumin, creatinine, BUN. Model 4 was further adjusted for heart failure

Test for trend based on weighted median blood selenium levels for each quantile

Models were adjusted for age, gender, race, education level, PIR, serum cotinine, BMI, physical activity, alcohol, hypertension, hyperuricemia, TC, HDL-C, albumin, creatinine, BUN, heart failure/HbA1c.

### Associations of Blood Selenium Levels with Heart Failure

As presented in Table 3, overall, we found a negative association between blood selenium levels and the risk of heart failure in all three models, with the risk of heart failure decreasing as blood selenium levels increased (*P* for trend =0.021). In model 3, compared to participants in the first quartile, blood selenium levels were significantly negatively associated with the risk of heart failure in participants in the second, third and fourth quartiles (Q2, OR=0.677, 95% CI: 0.471, 0.974) (Q3, OR=0.609, 95% CI: 0.426, 0.870) (Q4, OR=0.653, 95% CI. 0.443, 0.961). For every 10ug/L increase in blood selenium levels, the risk of heart failure decreased by an average of 5.0% (95% CI: 0.1%, 9.8%). The RCS regression showed a negative dose-response relationship between blood selenium levels and risk of heart failure (*P*_overall_ =0.015, *P*_non-linear_=0.053, Fig. 2B).

**Table 3:** Associations of blood selenium levels with heart failure

Selenium	Q1 (<176.96μg/L)	Q2 (176.96-191.62μg/L)	Q3 (191.62-207.22μg/L)	Q4 (>207.22μg/L)	*P* for trend	per 10μg/L increment
Model1	ref(1.000)	**0.629(0.451,0.876)**	**0.568(0.406,0.796)**	**0.582(0.395,0.856)**	**0.005**	**0.926(0.878,0.978)**
Model2	ref(1.000)	**0.622(0.437,0.886)**	**0.557(0.398,0.781)**	**0.568(0.376,0.857)**	**0.005**	**0.926(0.875,0.979)**
Model3	ref(1.000)	**0.677(0.471,0.974)**	**0.609(0.426,0.870)**	**0.653(0.443,0.961)**	**0.021**	**0.950(0.902,0.999)**

OR(95% CI) were calculated with the multivariate logistic regression model; Bold formatting indicated statistically significant differences

Model 1 was adjusted for demographic characteristics, including age, gender, race, education level and PIR. Model 2 was further adjusted for serum cotinine, BMI, physical activity, alcohol, hypertension. Model 3 was further adjusted for hyperuricemia, TC, HDL-C, albumin, creatinine, BUN, HbA1c

Test for trend based on weighted median blood selenium levels for each quantile

### Subgroup Analysis

As shown in Table 4, in model 1 and model 2, blood selenium levels were negatively associated with the risk of heart failure whether grouped by diabetes and poor glycemic control. However, after further adjustment for hyperuricemia, TC, HDL-C, albumin, creatinine, BUN, and HbA1c, this association was no longer significant. Model 3 showed that compared to the first quartile, the risk of heart failure was lowest in diabetic patients at the second quartile (OR=0.597, 95% CI: 0.373, 0.955), in non-diabetic patients at the third quartile level (OR=0.567, 95% CI: 0.327, 0.983), and in poor glycemic control participants at the third quartile level (OR=0.540, 95% CI:0.286,0.987). Furthermore, the association between blood selenium levels and heart failure was more pronounced in participants with poor glycemic control, rather than diabetic patients (*P* for interaction = 0.085).

**Table 4:** Associations of blood selenium levels with heart failure in diabetes (yes or no) and poor glycemic control (yes or no)

	Selenium				*P* for trend	per 10μg/L increment	*P* for interaction
	Q1 (<176.96μg/L)	Q2 (176.96-191.62μg/L)	Q3 (191.62-207.22μg/L)	Q4 (>207.22μg/L)			
Model1							
Diabetes							0.579
No	ref(1.000)	0.658(0.406,1.065)	**0.503(0.291,0.870)**	**0.546(0.345,0.864)**	**0.007**	**0.915(0.846,0.989)**	
Yes	ref(1.000)	**0.588(0.385,0.897)**	0.631(0.380,1.048)	0.562(0.297,1.065)	0.088	**0.925(0.855,1.000)**	
Poor glycemic control							**0.036**
No	ref(1.000)	0.705(0.481,1.034)	**0.582(0.369,0.918)**	0.663(0.432,1.020)	**0.044**	**0.942(0.889,0.999)**	
Yes	ref(1.000)	**0.548(0.318,0.920)**	**0.441(0.246,0.762)**	**0.393(0.224,0.668)**	**<0.001**	**0.877(0.812,0.944)**	
Model2							
Diabetes							0.657
No	ref(1.000)	0.672(0.404,1.115)	**0.505(0.289,0.880)**	**0.550(0.334,0.906)**	**0.011**	**0.918(0.846,0.995)**	
Yes	ref(1.000)	**0.555(0.355,0.867)**	0.606(0.361,1.017)	0.541(0.285,1.030)	0.075	**0.924(0.854,0.999)**	
Poor glycemic control							**0.065**
No	ref(1.000)	0.686(0.458,1.028)	**0.566(0.359,0.894)**	0.640(0.405,1.010)	**0.038**	**0.940(0.885,0.998)**	
Yes	ref(1.000)	0.605(0.347,1.030)	**0.501(0.276,0.880)**	**0.457(0.257,0.791)**	**0.003**	**0.900(0.832,0.969)**	
Model3							
Diabetes							0.615
No	ref(1.000)	0.758(0.452,1.273)	**0.567(0.327,0.983)**	0.679(0.414,1.112)	0.068	0.953(0.888,1.023)	
Yes	ref(1.000)	**0.597(0.373,0.955)**	0.646(0.368,1.134)	0.600(0.333,1.081)	0.115	0.940(0.871,1.016)	
Poor glycemic control							**0.085**
No	ref(1.000)	0.753(0.494,1.147)	0.641(0.403,1.020)	0.764(0.498,1.172)	0.154	0.969(0.920,1.020)	
Yes	ref(1.000)	0.695(0.389,1.213)	**0.540(0.286,0.987)**	0.569(0.307,1.031)	**0.040**	0.927(0.853,1.002)	

OR(95% CI) were calculated with the multivariate logistic regression model; Bold formatting indicated statistically significant differences

Model 1 was adjusted for demographic characteristics, including age, gender, race, education level and PIR. Model 2 was further adjusted for serum cotinine, BMI, physical activity, alcohol, hypertension. Model 3 was further adjusted for hyperuricemia, TC, HDL-C, albumin, creatinine, BUN, HbA1c

Test for trend based on weighted median blood selenium levels for each quantile

Test for interaction between blood selenium levels (continuous) and covariates

Subgroup analysis showed the association between diabetes and high blood selenium levels was more obvious in participants with tobacco exposure (*P* for interaction=0.082), alcohol consumption (*P* for interaction=0.003) and normal total cholesterol (*P* for interaction<0.001). Table S1 showed that compared to the first quartile, the risk of diabetes increased significantly when the blood selenium levels were in the fourth quartile for participants of those aged 20-59, those aged≥60, male, female, high school education level or below, above high school education level, those with PIR≥3.5, without tobacco exposure, BMI≥30.0, physically inactive, alcohol consumption, hypertensive, non-hyperuricemic, normal total cholesterol, and non-heart failure (Table S1).

Table S2 showed that the association between heart failure and blood selenium levels was more significant in those with normal total cholesterol (*P* for interaction<0.10) (Table S2). Participants with blood selenium levels in the first quartile group were associated with an increased risk of heart failure among those aged 60 years or older, those with less than high school education, PIR ≥3.5, without tobacco exposure, BMI≥30.0, alcohol consumption or not, hypertension or not, non-hyperuricemic, and those with normal and high total cholesterol. In contrast, after adjusting for confounders, participants in the second or third quartile of blood selenium had the lowest risk of heart failure.

### Sensitivity Analysis

The results of the sensitivity analysis showed the robustness of our findings in the original model (Table S3). We sequentially excluded participants taking selenium supplements, participants aged 80 years and older, those with missing PIR data, those with missing alcohol consumption, those currently using insulin, and those currently taking glucose-lowering medication, and blood selenium levels were all positively associated with the risk of diabetes. Then, we sequentially excluded participants with blood selenium levels greater than 400, and participants aged 80 years and older, all of whom had a negative association between blood selenium levels and heart failure (Q2, Q3, Q4 vs Q1, respectively) (Table S4).

## Discussion

This study, leveraging data from NHANES 2010-2020 pre, was the first to probe the relationship between blood selenium levels with diabetes and heart failure over the past decade. It aimed to comprehensively clarify the association across different subgroups to identify at-risk groups and provide targeted prevention strategies for the development of diabetes and heart failure, as well as providing a reference for subsequent studies. Our analysis revealed a positive association between high blood selenium levels and diabetes, particularly among individuals with tobacco exposure, alcohol consumption, and normal total cholesterol levels. Conversely, low blood selenium levels were associated with heart failure, indicating that both excessively high and low selenium levels may have adverse health consequences. Furthermore, the association between blood selenium levels and heart failure was more pronounced in participants with poor glycemic control, rather than diabetic patients.

This study found a positive association between high blood selenium levels and diabetes, which aligns with the results reported by Moon S et al. [9] and Vinceti M et al. [28]. Another study utilizing NHANES 2003-2004 data demonstrated that elevated serum selenium concentrations were linked to a higher prevalence of diabetes, as well as increased fasting blood glucose and glycohemoglobin levels [29]. A meta-analysis indicated that compared to an intake level of less than 23 μg/day, 50 μg/day and 75 μg/day were associated with a 50% (10%, 90%) and 90% (40%, 170%) higher risk of diabetes, respectively [30]. A systematic review and dose-response meta-analysis of non-experimental studies found that compared to 90 μg/L, blood/plasma/serum selenium concentrations of 160 μg/L associated with an increased risk of diabetes (OR=1.96, 95% CI: 1.27, 3.03) [28]. Excessive accumulation of selenium in the body may cause oxidative stress by interfering with cellular redox processes [31, 32], which may directly or indirectly disrupt insulin metabolism [33], leading to insulin resistance and an increase the risk of diabetes mellitus. Moreover, it may also affect glucose metabolism by impacting crucial regulators of glycolysis and gluconeogenesis [34]. However, no significant association between blood selenium levels and diabetes was observed in the study by Barbara R Cardoso et al. [10]. This discrepancy may be due to different confounding factors masking the correlation.

Subgroup analysis revealed that the association between high blood selenium levels and diabetes was more pronounced in participants with tobacco exposure and alcohol consumption, corroborating findings from prior studies. The presence of harmful substances such as arsenic in tobacco may chemically interact with selenium and impact its expression and metabolism [35]. In an animal research, excessive alcohol consumption was observed to reduce selenium absorption and affected liver expression of selenoproteins [36]. Interestingly, our findings also indicated that the association between high blood selenium levels and diabetes was more pronounced in those with normal total cholesterol, similar to the results of a Mendelian randomization study [37]. The plausible hypothesis was that elevated total cholesterol masked the deleterious effects of aberrant blood selenium concentrations, or that anomalous selenium levels played a role in lipid metabolism. However, the precise mechanisms warranted further exploration.

We observed that low blood selenium levels were associated with heart failure, which is consistent with the results of the BIOSTAT-CHF cohort study [38] and Yang L et al. [39]. A meta-analysis showed the standard mean deviation (SMD) of selenium levels in heart failure patients were significantly lower than the healthy control group (SMD=-0.98, 95% CI: -1.34, -0.62) [39]. In vitro experiments demonstrated that selenium supplementation increased cardiac superoxide dismutase 2 (SOD2), glutathione peroxidase (Gpx) and glutathione (GSH) levels and SOD activity, while reducing apoptosis [40]. Furthermore, selenium insufficiency in cultured human cardiomyocytes potentially compromised mitochondrial efficiency and oxidative phosphorylation and amplified intracellular reactive oxygen species, intensifying oxidative stress and inflammation [13].

The association between blood selenium levels and heart failure was more pronounced in participants with poor glycemic control, rather than diabetic patients, which stressed the importance of controlling blood glucose levels for the whole population, especially for diabetic patients. People with poor blood glucose control are more likely to experience microcirculation disorders, oxidative stress, and inflammatory damage. Such conditions could compromise the structural integrity of myocardial cells, rendering them more vulnerable to diminished blood selenium concentrations [41, 42].

However, it is also possible that elevated or reduced selenium levels could be both a cause and an outcome of the disease that further aggravates the condition [43]. The exact mechanism behind this relationship needs to be confirmed through higher hierarchy of evidences. In future research, it is important to consider more detailed disease staging and account for factors such as medication use, disease history, comorbidities, in order to obtain more accurate evidence. Assessment of risk factors for diabetes and heart failure should include consideration of blood selenium levels along with traditional risk factors. For individuals with sufficient selenium levels, blind selenium supplementation is not recommended and should only be done under medical guidance.

## Limitations and Strengths

This study has several strengths. Firstly, we took advantage of NHANES 2011–2020 pre data, with weights taken into account in the analysis, allowing for a more accurate representation of the general US adult population. This provides a valuable reference point for assessing the relationship between blood selenium levels and diabetes and heart failure in the general US adult population over the past decade. Secondly, we conducted detailed subgroup analysis and introduce interaction terms to clarify high-risk populations. Thirdly, considering both heart failure and blood glucose control simultaneously may provide a more accurate reflection of the actual situation.

There are several limitations to this study. Firstly, the cross-sectional design of the study limited the ability to interpret the causal relationship between blood selenium levels and diabetes and heart failure. Secondly, the use of self-reported heart failure may have biased the results and the type and severity of heart failure were not specifically studied. Thirdly, the focus was solely on the living diseased population, omitting the deceased, which could lead to survivorship bias. Fourthly, pertained exclusively to the general American adults, potentially restricting the generalizability of our findings. Finally, other potential confounding factors such as dietary intake and medication usage were not considered in the analysis.

## Conclusion

The findings of the study suggested that there may be a positive correlation between high blood selenium levels and diabetes, as well as a negative association between low blood selenium levels and heart failure, indicating that both high and low levels of selenium may have negative effects on the body. Furthermore, the association between blood selenium levels and heart failure was more pronounced in participants with poor glycemic control, rather than diabetic patients. Based on these results, it is recommended that individuals should avoid tobacco exposure, reduce alcohol consumption, regularly monitor their blood glucose levels, and maintain appropriate blood selenium levels in order to minimize the risk factors associated with diabetes and heart failure.

### Supplementary Information

ESM 1

## Data Availability

Publicly available datasets were analyzed in this study. This data can be found in NHANES’s official website(https://www.cdc.gov/nchs/nhanes/index.htm).

## References

[1] Sun H, Saeedi P, Karuranga S, Pinkepank M, Ogurtsova K, Duncan BB, Stein C, Basit A, Chan JCN, Mbanya JC (2022). IDF Diabetes Atlas: Global, regional and country-level diabetes prevalence estimates for 2021 and projections for 2045. Diabetes Res Clin Pract.

[2] GBD (2019). Diabetes in the Americas Collaborators (2022) Burden of diabetes and hyperglycaemia in adults in the Americas, 1990-2019: a systematic analysis for the Global Burden of Disease Study 2019. Lancet Diabetes Endocrinol.

[3] Pop-Busui R, Januzzi JL, Bruemmer D, Butalia S, Green JB, Horton WB, Knight C, Levi M, Rasouli N, Richardson CR (2022). Heart Failure: An Underappreciated Complication of Diabetes. A Consensus Report of the American Diabetes Association. Diabetes Care.

[4] Yang HH, Li FR, Chen ZK, Zhou MG, Xie LF, Jin YY, Li ZH, Chen GC (2023). Duration of Diabetes, Glycemic Control, and Risk of Heart Failure Among Adults With Diabetes: A Cohort Study. J Clin Endocr Metab.

[5] McDonagh TA, Metra M, Adamo M, Gardner RS, Baumbach A, Bohm M, Burri H, Butler J, Celutkien J, Chioncel O (2022). 2021 ESC Guidelines for the diagnosis and treatment of acute and chronic heart failure Developed by the Task Force for the diagnosis and treatment of acute and chronic heart failure of the European Society of Cardiology (ESC) With the special contribution of the Heart Failure Association (HFA) of the ESC. Eur J Heart Fail.

[6] James SL, Abate D, Abate KH, Abay SM, Abbafati C, Abbasi N, Abbastabar H, Abd-Allah F, Abdela J, Abdelalim A (2018). Global, regional, and national incidence, prevalence, and years lived with disability for 354 diseases and injuries for 195 countries and territories, 1990-2017: a systematic analysis for the Global Burden of Disease Study 2017. Lancet.

[7] Rayman MP (2020). The importance of selenium to human health. Lancet.

[8] Filippini T, Urbano T, Grill P, Malagoli C, Ferrari A, Marchesi C, Natalini N, Michalke B, Vinceti M (2023) Human serum albumin-bound selenium (Se-HSA) in serum and its correlation with other selenium species. J Trace Elem Med Bio 79. 10.1016/j.jtemb.2023.127266 10.1016/j.jtemb.2023.127266 37499550

[9] Moon S, Chung HS, Yu JM, Yoo HJ, Park JH, Kim DS, Park YK, Yoon SN (2019). Association between serum selenium level and the prevalence of diabetes mellitus in US population. J Trace Elem Med Bio.

[10] Cardoso BR, Braat S, Graham RM (2021) Selenium Status Is Associated With Insulin Resistance Markers in Adults: Findings From the 2013 to 2018 National Health and Nutrition Examination Survey (NHANES). Front Nutr 8. 10.3389/fnut.2021.696024 10.3389/fnut.2021.696024 PMC8273176 34262926

[11] Tan QH, Huang YQ, Liu XC, Liu L, Lo K, Chen JY, Feng YQ (2021). A U-Shaped Relationship Between Selenium Concentrations and All-Cause or Cardiovascular Mortality in Patients With Hypertension. Front Cardiovasc Med.

[12] Liao XL, Wang ZH, Liang XN, Liang J, Wei XB, Wang SH, Guo WX (2020). The Association of Circulating Selenium Concentrations with Diabetes Mellitus. Diabet Metab Synd Ob.

[13] Bomer N, Beverborg NG, Hoes MF, Streng KW, Vermeer M, Dokter MM, IJmker J, Anker SD, Cleland JGF, Hillege HL (2020). (2020) Selenium and outcome in heart failure. Eur J Heart Fail.

[14] National Center for Health Statistics, Centers for Disease Control and Prevention. NHANES questionnaires, datasets, and related documentation. https://wwwn.cdc.gov/nchs/nhanes/default.aspx. (accessed May 7 2023).

[15] Akinbami LJCT, Davy O, Ogden CL, Fink S, Clark J, Riddles MK, Mohadjer LK (2022). National Health and Nutrition Examination Survey, 2017-March 2020 Prepandemic file: Sample Design, Estimation, and Analytic Guidelines. Vital Health Stat.

[16] Fairweather-Tait SJ, Bao YP, Broadley MR, Collings R, Ford D, Hesketh JE, Hurst R (2011). Selenium in Human Health and Disease. Antioxid Redox Sign.

[17] National Center for Health Statistics, Centers for Disease Control and Prevention. 2015-2016 Data Documentation, Codebook, and Frequencies: Lead, Cadmium, Total Mercury, Selenium & Manganese – Blood. https://wwwn.cdc.gov/Nchs/Nhanes/2015-2016/PBCD_I.htm. (accessed October 4 2023).

[18] Assoc AD (2020). Classification and Diagnosis of Diabetes: Standards of Medical Care in Diabetes-2020. Diabetes Care.

[19] Ciardullo S, Cannistraci R, Mazzetti S, Mortara A, Perseghin G (2022). Twenty-year trends in heart failure among US adults, 1999-2018: The growing impact of obesity and diabetes. Int J Cardiol.

[20] Whelton PK, Carey RM, Aronow WS (2018). 2017 ACC/AHA/AAPA/ABC/ACPM/AGS/APhA/ASH/ASPC/NM CNA Guideline for the Prevention, Detection, Evaluation, and Management of High Blood Pressure in Adults A Report of the American College of Cardiology/American Heart Association Task Force on Clinical Practice Guidelines. Hypertension.

[21] Li SY, Ding J, Sun XX, Feng L, Zhou WH, Gui Z, Mao JF (2023) Selenium Concentration Is Positively Associated with Triglyceride-Glucose Index and Triglyceride Glucose-Body Mass Index in Adults: Data from **N**HANES 2011-2018. Biol Trace Elem Res. 10.1007/s12011-023-03684-2 Online ahead of print 10.1007/s12011-023-03684-2 PMC10764531 37145256

[22] Ma YDY, Hu Q, Yang DH, Zhao YD, Bai JJ, Mubarik S, Yu CH (2022) Combined exposure to multiple metals on serum uric acid in NHANES under three statistical models. Chemosphere 301. 10.1016/j.chemosphere.2022.134416 10.1016/j.chemosphere.2022.134416 35490746

[23] Aggarwal R, Bhatt DL, Rodriguez F, Yeh RW, Wadhera RK (2022). Trends in Lipid Concentrations and Lipid Control Among US Adults, 2007-2018. Jama-J Am Med Assoc.

[24] Pra ADAP (2022). 6. Glycemic Targets: Standards of Medical Care in Diabetes-2022. Diabetes Care.

[25] Han TS, Zhang S, Duan W, Ren XH, Wei CB, Sun CH, Li Y (2019). Eighteen-year alcohol consumption trajectories and their association with risk of type 2 diabetes and its related factors: the China Health and Nutrition Survey. Diabetologia.

[26] Desquilbet L, Mariotti F (2010). Dose-response analyses using restricted cubic spline functions in public health research. Stat Med.

[27] Liu CQ, Cao GX, Li JY et al (2023) Effect of long-term exposure to PM2.5 on the risk of type 2 diabetes and arthritis in type 2 diabetes patients: Evidence from a national cohort in China. Environ Int 171. 10.1016/j.envint.2023.107741 10.1016/j.envint.2023.107741 36628860

[28] Vinceti M, Filippini T, Wise LA, Rothman KJ (2021) A systematic review and dose-response meta-analysis of exposure to environmental selenium and the risk of type 2 diabetes in nonexperimental studies. Environ Res 197. 10.1016/j.envres.2021.111210 10.1016/j.envres.2021.111210 33895112

[29] Bleys J, Navas-Acien A, Guallar E (2007). Serum selenium and diabetes in US adults. Diabetes Care.

[30] Vinceti M, Filippini T, Rothman KJ (2018). Selenium exposure and the risk of type 2 diabetes: a systematic review and meta-analysis. Eur J Epidemiol.

[31] Urbano T, Filippini T, Wise LA, Sucato S, Polledri E, Malavolti M, Fustinoni S, Michalke B, Vinceti M (2023) Selenium exposure and urinary 8-oxo-7,8-dihydro-2'-deoxyguanosine: Major effects of chemical species and sex. Sci Total Environ 870. 10.1016/j.scitotenv.2023.161584 10.1016/j.scitotenv.2023.161584 36702271

[32] Alharithy M, Alafif N (2023) Association of Selenium Intake and Selenium Concentrations with Risk of Type 2 Diabetes in Adults: A Narrative Review. Metabolites 13(6). 10.3390/metabo13060767 10.3390/metabo13060767 PMC10305237 37367924

[33] Murano K, Ogino H, Okuno T, Arakawa T, Ueno H (2018). Role of Supplementary Selenium on the Induction of Insulin Resistance and Oxidative Stress in NSY Mice Fed a High Fat Diet. Biol Pharm Bull.

[34] Casanova P, Monleon D (2023) Role of selenium in type 2 diabetes, insulin resistance and insulin secretion. World. J Diabetes 14(3). 10.4239/wjd.v14.i3.147 10.4239/wjd.v14.i3.147 PMC10075028 37035226

[35] Ali W, Zhang H, Junaid M, Mao K, Xu N, Chang CY, Rasool A, Aslam MW, Ali J, Yang ZG (2021). Insights into the mechanisms of arsenic-selenium interactions and the associated toxicity in plants, animals, and humans: A critical review. Crit Rev Env Sci Tec.

[36] Ojeda ML, Rua RM, Murillo ML, Carreras O, Nogales F (2015). Binge Drinking During Adolescence Disrupts Se Homeostasis and Its Main Hepatic Selenoprotein Expression. Alcohol Clin Exp Res.

[37] Rath AA, Lam HS, Schooling CM (2021). Effects of selenium on coronary artery disease, type 2 diabetes and their risk factors: a Mendelian randomization study. Eur J Clin Nutr.

[38] Weening EH, Al-Mubarak AA, Dokter MM et al (2023) Sexual dimorphism in selenium deficiency is associated with metabolic syndrome and prevalence of heart disease. Cardiovasc Diabetol 22(1). 10.1186/s12933-022-01730-2 10.1186/s12933-022-01730-2 PMC9838024 36635707

[39] Yang L, Qi M, Du X, Xia Z, Fu G, Chen X, Liu Q, Sun N, Shi C, Zhang R (2022) Selenium concentration is associated with occurrence and diagnosis of three cardiovascular diseases: A systematic review and meta-analysis. J Trace Elem Med Bio 70. 10.1016/j.jtemb.2021.126908 10.1016/j.jtemb.2021.126908 34902677

[40] Shengyu C, Yinhua L, Yuanhong L, Jinbo Z, Can F, Hao X, Changjiang Z (2022) Selenium alleviates heart remodeling through Sirt1/AKT/GSK-3? pathway. Int Immunopharmacol 111. 10.1016/j.intimp.2022.109158 10.1016/j.intimp.2022.109158 35987147

[41] Marfella R, Di Filippo C, Portoghese M, Ferraraccio F, Rizzo MR, Siniscalchi M, Musacchio E, D'Amico M, Rossi F, Paolisso G (2009). Tight Glycemic Control Reduces Heart Inflammation and Remodeling During Acute Myocardial Infarction in Hyperglycemic Patients. J Am Coll Cardiol.

[42] Yang CD, Aihemaiti M, Wei J, Chen JW, Shu XY, Ding FH, Shen WF, Lu L, Zhang RY, Pan WQ (2023). HbA1c level is associated with the development of heart failure with recovered ejection fraction in hospitalized heart failure patients with type 2 diabetes. Int J Cardiol.

[43] Steinbrenner H, Duntas LHH, Rayman MPP (2022) The role of selenium in type-2 diabetes mellitus and its metabolic comorbidities. Redox Biol 50. 10.1016/j.redox.2022.102236 10.1016/j.redox.2022.102236 PMC8844812 35144052

