# Supplementary material for: Association of Blood Selenium Levels with Diabetes and Heart Failure in American General Adults: a Cross-sectional Study of NHANES 2011–2020 pre
Source: Biol Trace Elem Res. 2023 Nov 23;202(8):3413–24. doi: 10.1007/s12011-023-03933-4 (PMC11144148; doi:10.1007/s12011-023-03933-4)
Supplement: Supplementary file 1 — ESM 1 [file 12011_2023_3933_MOESM1_ESM.docx]

Additional file1

TableS1. Subgroup analysis of the associations of blood selenium levels with diabetes

|  | Selenium | | | | *P* for trend | per 10μg/L  increment | *P* for  interaction |  |
| --- | --- | --- | --- | --- | --- | --- | --- | --- |
|  | Q1 | Q2 | Q3 | Q4 |  |  |  |  |
| **Age** |  |  |  |  |  |  | 0.112 |  |
| 20-59 | ref.(1.000) | 0.993(0.742,1.330) | 1.020(0.788,1.320) | **1.469(1.076,2.004)** | **0.013** | **1.065(1.017,1.115)** |  |  |
| ≥60 | ref.(1.000) | 1.034(0.821,1.303) | 1.143(0.865,1.510) | **1.385(1.048,1.831)** | **0.023** | **1.027(1.000,1.054)** |  |  |
| **Gender** |  |  |  |  |  |  | 0.265 |  |
| Male | ref.(1.000) | 1.142(0.837,1.557) | 1.266(0.933,1.719) | **1.496(1.109,2.018)** | **0.008** | **1.051(1.019,1.085)** |  |  |
| Female | ref.(1.000) | 0.973(0.753,1.258) | 0.918(0.691,1.218) | **1.415(1.074,1.865)** | **0.030** | 1.026(0.994,1.058) |  |  |
| **Education level** |  |  |  |  |  |  | 0.558 |  |
| High school or Less | ref.(1.000) | 1.087(0.83,1.424) | 1.119(0.843,1.484) | **1.331(1.011,1.752)** | **0.048** | 1.029(0.991,1.068) |  |  |
| Above High school | ref.(1.000) | 1.013(0.767,1.338) | 1.091(0.804,1.481) | **1.542(1.113,2.135)** | **0.007** | **1.049(1.017,1.083)** |  |  |
| **PIR** |  |  |  |  |  |  | 0.147 |  |
| ≤1.30 | ref.(1.000) | 1.052(0.776,1.427) | 1.120(0.831,1.509) | 1.423(0.996,2.031) | 0.051 | **1.073(1.034,1.115)** |  |  |
| 1.31-3.49 | ref.(1.000) | 0.885(0.646,1.212) | 1.116(0.736,1.692) | 1.274(0.878,1.849) | 0.128 | 1.025(0.982,1.069) |  |  |
| ≥3.50 | ref.(1.000) | 1.195(0.802,1.782) | 1.086(0.718,1.642) | **1.671(1.125,2.481)** | **0.016** | **1.041(1.002,1.082)** |  |  |
| **Serum Cotinine** |  |  |  |  |  |  | **0.082** |  |
| <1.00 ng/ml | ref.(1.000) | 1.087(0.865,1.366) | 1.161(0.924,1.459) | **1.473(1.165,1.861)** | **0.002** | **1.035(1.006,1.065)** |  |  |
| ≥1.00 ng/ml | ref.(1.000) | 0.897(0.607,1.326) | 0.936(0.610,1.437) | 1.380(0.919,2.074) | 0.133 | **1.076(1.009,1.147)** |  |  |
| **BMI** |  |  |  |  |  |  | 0.124 |  |
| <25.00 | ref.(1.000) | 1.019(0.663,1.566) | 0.990(0.562,1.746) | 1.274(0.718,2.257) | 0.463 | 1.024(0.969,1.082) |  |  |
| 25.00-29.99 | ref.(1.000) | 0.971(0.682,1.384) | 1.075(0.757,1.527) | 1.393(0.927,2.092) | 0.104 | **1.056(1.014,1.099)** |  |  |
| ≥30.00 | ref.(1.000) | 1.037(0.787,1.366) | 1.112(0.840,1.471) | **1.506(1.148,1.976)** | **0.003** | 1.038(0.994,1.084) |  |  |
| **Physical activity** |  |  |  |  |  |  | 0.111 |  |
| No | ref.(1.000) | 1.275(0.901,1.805) | 1.224(0.900,1.666) | **1.714(1.282,2.293)** | **0.001** | **1.066(1.020,1.113)** |  |  |
| Yes | ref.(1.000) | 0.906(0.715,1.147) | 1.013(0.766,1.339) | 1.302(0.991,1.709) | **0.039** | 1.029(0.998,1.060) |  |  |
| **Alcohol** **consumption** |  |  |  |  |  |  | **0.003** |  |
| No | ref.(1.000) | 0.992(0.768,1.281) | 1.073(0.792,1.455) | 1.227(0.900,1.672) | 0.169 | 1.017(0.988,1.047) |  |  |
| Yes | ref.(1.000) | 1.105(0.827,1.475) | 1.122(0.833,1.512) | **1.638(1.212,2.214)** | **0.002** | **1.073(1.036,1.111)** |  |  |
| **Hypertension** |  |  |  |  |  |  | 0.464 |  |
| No | ref.(1.000) | 0.914(0.632,1.324) | 1.045(0.743,1.471) | 1.313(0.887,1.942) | 0.129 | **1.043(1.000,1.088)** |  |  |
| Yes | ref.(1.000) | 1.129(0.927,1.374) | 1.105(0.866,1.41) | **1.504(1.182,1.913)** | **0.003** | **1.037(1.007,1.068)** |  |  |
| **Hyperuricemia** |  |  |  |  |  |  | 0.768 |  |
| No | ref.(1.000) | 1.033(0.828,1.288) | 1.134(0.908,1.416) | **1.512(1.143,1.999)** | **0.003** | **1.040(1.008,1.073)** |  |  |
| Yes | ref.(1.000) | 1.084(0.712,1.652) | 1.024(0.688,1.525) | 1.247(0.866,1.795) | 0.294 | 1.041(0.991,1.094) |  |  |
| **High total cholesterol** |  |  |  |  |  |  | **<0.001** |  |
| No | ref.(1.000) | 1.026(0.816,1.289) | 1.065(0.824,1.377) | **1.530(1.216,1.926)** | **0.001** | **1.060(1.029,1.092)** |  |  |
| Yes | ref.(1.000) | 0.981(0.667,1.444) | 1.068(0.680,1.677) | 1.201(0.865,1.667) | 0.193 | 1.006(0.974,1.040) |  |  |
| **Heart Failure** |  |  |  |  |  |  | 0.975 |  |
| No | ref.(1.000) | 1.044(0.845,1.290) | 1.107(0.920,1.332) | **1.458(1.169,1.818)** | **0.001** | **1.042(1.015,1.070)** |  |  |
| Yes | ref.(1.000) | 1.215(0.719,2.057) | 1.299(0.722,2.346) | 1.658(0.909,3.054) | 0.096 | 1.067(0.992,1.150) |  |  |

Note: OR(95% CI) were calculated with the multivariate logistic regression model.

The associations were adjusted for age, gender, race, education level, PIR, serum cotinine, BMI, physical activity, alcohol, hypertension, hyperuricemia, TC, HDL-C, albumin, creatinine, BUN, heart failure.

Test for trend based on weighted median blood selenium levels for each quantile.

Test for interaction between blood selenium levels (continuous) and covariates.

Additional file2

TableS2. Subgroup analysis of the associations of blood selenium levels with heart failure

|  | Selenium | | | | *P* for  trend | per 10μg/L  increment | *P* for  interaction |
| --- | --- | --- | --- | --- | --- | --- | --- |
|  | Q1 | Q2 | Q3 | Q4 |  |  |  |
| **Age** |  |  |  |  |  |  | 0.203 |
| 20-59 | ref(1.000) | 0.639(0.356,1.147) | 0.631(0.335,1.191) | 0.827(0.418,1.634) | 0.520 | 0.977(0.854,1.118) |  |
| ≥60 | ref(1.000) | 0.676(0.425,1.074) | **0.550(0.320,0.945)** | **0.567(0.337,0.954)** | **0.027** | **0.930(0.874,0.989)** |  |
| **Gender** |  |  |  |  |  |  | 0.804 |
| Male | ref(1.000) | 0.627(0.384,1.023) | 0.603(0.359,1.013) | 0.561(0.312,1.010) | 0.053 | 0.961(0.888,1.04) |  |
| Female | ref(1.000) | 0.735(0.415,1.301) | 0.601(0.333,1.083) | 0.786(0.457,1.352) | 0.252 | 0.936(0.87,1.007) |  |
| **Education level** |  |  |  |  |  |  | 0.645 |
| High school or Less | ref(1.000) | **0.599(0.391,0.920)** | **0.547(0.348,0.859)** | **0.534(0.314,0.910)** | **0.012** | 0.932(0.853,1.019) |  |
| Above High school | ref(1.000) | 0.739(0.420,1.299) | 0.686(0.395,1.194) | 0.799(0.439,1.455) | 0.468 | 0.967(0.904,1.034) |  |
| **PIR** |  |  |  |  |  |  | 0.420 |
| ≤1.30 | ref(1.000) | 0.994(0.624,1.583) | 0.826(0.477,1.429) | 0.730(0.407,1.310) | 0.254 | 0.958(0.887,1.034) |  |
| 1.31-3.49 | ref(1.000) | 0.709(0.440,1.144) | 0.565(0.313,1.019) | 0.747(0.444,1.256) | 0.206 | 0.957(0.903,1.013) |  |
| ≥3.50 | ref(1.000) | **0.315(0.117,0.852)** | 0.444(0.192,1.028) | **0.382(0.159,0.917)** | 0.054 | 0.894(0.744,1.073) |  |
| **Serum Cotinine** |  |  |  |  |  |  | 0.459 |
| <1.00 ng/ml | **ref(1.000)** | **0.598(0.364,0.981)** | **0.604(0.369,0.986)** | **0.613(0.380,0.987)** | 0.054 | 0.954(0.897,1.015) |  |
| ≥1.00 ng/ml | ref(1.000) | 1.037(0.554,1.944) | 0.652(0.330,1.285) | 0.879(0.382,2.023) | 0.537 | 0.951(0.841,1.076) |  |
| **BMI** |  |  |  |  |  |  | 0.172 |
| <25.00 | ref(1.000) | 1.800(0.796,4.071) | 0.592(0.209,1.679) | 0.524(0.193,1.426) | 0.135 | 0.936(0.83,1.055) |  |
| 25.00-29.99 | **ref(1.000)** | 0.692(0.362,1.324) | 0.578(0.279,1.199) | 0.522(0.259,1.055) | 0.058 | 0.956(0.866,1.054) |  |
| ≥30.00 | **ref(1.000)** | **0.471(0.299,0.739)** | **0.602(0.365,0.993)** | 0.691(0.428,1.114) | 0.194 | 0.953(0.899,1.011) |  |
| **Physical activity** |  |  |  |  |  |  | 0.543 |
| No | **ref(1.000)** | 0.663(0.402,1.093) | 0.595(0.354,1.003) | 0.676(0.386,1.183) | 0.126 | 0.953(0.896,1.013) |  |
| Yes | **ref(1.000)** | 0.696(0.397,1.219) | 0.609(0.359,1.034) | 0.618(0.346,1.105) | 0.091 | 0.942(0.864,1.028) |  |
| **Alcohol** **consumption** |  |  |  |  |  |  | 0.694 |
| No | **ref(1.000)** | **0.489(0.301,0.793)** | 0.652(0.354,1.198) | 0.597(0.334,1.069) | 0.125 | 0.952(0.888,1.020) |  |
| Yes | **ref(1.000)** | 0.872(0.554,1.372) | **0.566(0.344,0.933)** | 0.687(0.410,1.152) | 0.067 | 0.944(0.875,1.018) |  |
| **Hypertension** |  |  |  |  |  |  | 0.186 |
| No | **ref(1.000)** | 0.718(0.320,1.609) | **0.207(0.066,0.652)** | 0.424(0.157,1.147) | **0.034** | 0.882(0.762,1.020) |  |
| Yes | **ref(1.000)** | **0.669(0.479,0.934)** | 0.740(0.515,1.064) | 0.719(0.458,1.130) | 0.172 | 0.962(0.908,1.019) |  |
| **Hyperuricemia** |  |  |  |  |  |  | 0.334 |
| No | ref(1.000) | **0.600(0.372,0.967)** | **0.565(0.381,0.838)** | **0.589(0.350,0.994)** | **0.034** | 0.927(0.857,1.002) |  |
| Yes | ref(1.000) | 0.811(0.466,1.410) | 0.680(0.390,1.183) | 0.781(0.413,1.478) | 0.395 | 0.979(0.909,1.055) |  |
| **High total cholesterol** |  |  |  |  |  |  | **0.077** |
| No | ref(1.000) | **0.579(0.369,0.908)** | 0.683(0.449,1.037) | **0.442(0.268,0.729)** | **0.003** | **0.920(0.854,0.991)** |  |
| Yes | ref(1.000) | 1.058(0.518,2.162) | **0.368(0.158,0.858)** | 1.294(0.623,2.688) | 0.686 | 0.996(0.953,1.042) |  |

Note: OR(95% CI) were calculated with the multivariate logistic regression model.

The associations were adjusted for age, gender, race, education level, PIR, serum cotinine, BMI, physical activity, alcohol, hypertension, hyperuricemia, TC, HDL-C, albumin, creatinine, BUN, HbA1c.

Test for trend based on weighted median blood selenium levels for each quantile.

Test for interaction between blood selenium levels (continuous) and covariates.

Additional file3

Table S3. Sensitivity analysis of the associations of blood selenium levels with diabetes

|  | Selenium | | | | *P* for trend | per 10μg/L  increment |  |
| --- | --- | --- | --- | --- | --- | --- | --- |
|  | Q1 | Q2 | Q3 | Q4 |  |  |  |
| Model1 | ref.(1.000) | 1.083(0.856,1.370) | 1.206(0.978,1.488) | **1.406(1.103,1.793)** | **0.005** | **1.042(1.009,1.075)** |  |
| Model2 | ref.(1.000) | 1.010(0.807,1.263) | 1.097(0.893,1.347) | **1.454(1.152,1.834)** | **0.002** | **1.042(1.013,1.072)** |  |
| Model3 | ref.(1.000) | 1.050(0.851,1.296) | 1.140(0.933,1.393) | **1.548(1.235,1.941)** | **0.000** | **1.045(1.017,1.074)** |  |
| Model4 | ref.(1.000) | 0.955(0.743,1.228) | 1.063(0.811,1.393) | **1.398(1.069,1.829)** | **0.011** | **1.035(1.007,1.064)** |  |
| Model5 | ref.(1.000) | 1.082(0.872,1.342) | 1.149(0.927,1.424) | **1.496(1.177,1.901)** | **0.002** | **1.038(1.011,1.066)** |  |
| Model6 | ref.(1.000) | 1.193(0.893,1.594) | 1.002(0.723,1.390) | **1.389(1.016,1.898)** | 0.083 | 1.039(0.999,1.080) |  |

Note: OR(95% CI) were calculated with the multivariate logistic regression model.

The associations were adjusted for age, gender, race, education level, PIR, serum cotinine, BMI, physical activity, alcohol, hypertension, hyperuricemia, TC, HDL-C, albumin, creatinine, BUN, heart failure.

Model 1 excluded participants taking selenium supplements; Model 2 excluded participants aged 80 years and over; Model 3 excluded participants with missing PIR data; Model 4 excluded participants with missing alcohol consumption; Model 5 excluded participants taking insulin now; Model 6 excluded participants Take diabetic pills to lower blood sugar now.

Test for trend based on weighted median blood selenium levels for each quantile.

Additional file4

Table S4. Sensitivity analysis of the associations of blood selenium levels with heart failure

|  | Selenium | | | | *P* for trend | per 10μg/L  increment |  |
| --- | --- | --- | --- | --- | --- | --- | --- |
|  | Q1 | Q2 | Q3 | Q4 |  |  |  |
| Model1 | ref.(1.000) | **0.675(0.468,0.973)** | **0.620(0.433,0.888)** | **0.669(0.455,0.984)** | **0.031** | 0.954(0.904,1.007) |  |
| Model2 | ref.(1.000) | **0.640(0.434,0.944)** | **0.577(0.389,0.857)** | **0.612(0.391,0.957)** | **0.022** | 0.939(0.880,1.003) |  |

Note: OR(95% CI) were calculated with the multivariate logistic regression model.

The associations were adjusted for age, gender, race, education level, PIR, serum cotinine, BMI, physical activity, alcohol, hypertension, hyperuricemia, TC, HDL-C, albumin, creatinine, BUN, HbA1c.

Model 1 excluded participants with blood selenium levels more than 400; Model 2 excluded participants aged 80 years and over.

Test for trend based on weighted median blood selenium levels for each quantile.
